# The New York Homœopathic Union

**Published:** 1894-05

**Authors:** Emma D. Wilcox

**Affiliations:** Secretary


### THE NEW YORK HOMŒOPATHIC UNION.

A regular meeting of the New York Homœopathic Union was held at 53 West 45th Street, January 18th, 1894, the President, Edmund Carleton, M. D., in the chair. Present: Drs. Allan, Baylies, Boyle, Campbell, Clark, Dillingham, Dyer, O'Brien, Powel, Wilcox, and Young.

Dr. Wilcox was chosen Secretary *pro tem.* Letters of regret were read from Drs. Fincke, Morgan, and Stanton.

Reading of *The Organon* was then taken up, Sections 231–240, inclusive. Section 234 at once provoked discussion. It reads as follows: Section 234. Those morbid states apparently without fever, which assume a particular type—that is to say, which return at fixed periods in the same patient (they do not manifest themselves, in general, either sporadically or epidemically)—all belong to the class of chronic diseases. The greater number of them depend upon a simple psoric affection, seldom complicated with syphilis, and they are combatted successfully by the same treatment. It is, however, sometimes necessary to have recourse to a very small dose of attenuated solution of Cinchona, for the purpose of completely extinguishing their intermittent form.

Dr. Baylies—Hahnemann says *sometimes* a dose of Cinchona. He does not say *often*. The symptoms may correspond and that drug be needed, even though not reckoned among the antipsorics.

Dr. Young—The intermittent form is often so intense as to need to be quelled before the latent psora will be shown.

Dr. Dyer—An intensity of partial symptoms shows an incomplete and latent case in which an antisporic would bring out the true conditions. In the one spoken of Hahnemann thinks that Cinchona would usually accomplish the result.

Dr. Baylies gave a case under psoric conditions with intense periodicity, where the antisporic had but temporary effect. He then selected an antiperiodic, which cured.

Sections 235–237 were then considered.

The President—If Hahnemann had written nothing else he would have bestowed a blessing upon mankind by these three paragraphs alone.

Dr. Young—Do you not think that attention to time of recurrence often misleading?

To illustrate, he gave a case where symptoms and time of recurrence pointed strongly to Eup-perf., but with no relief following. History showed chronic psora. Also vomiting between chill and heat, sour, not bitter. Lyc. cured.

Dr. Clark had had a similar experience.

The President—The choice between *Eupatorium-perfoliatum* and *Lycopodium* is sometimes exceedingly close in cases of intermittent fever, and this upon the score of pure symptomatology without regard to psora. Both have vomiting after the chill and before the fever, but the vomiting of Eupatorium is *bitter*, while that of Lycopodium is *sour*, as has been stated. In such cases that feature is decisive. The remedy given in accordance cures *every time*, the other *never*. Neither can be substituted for the other.

Dr. Young—If there is no improvement after the second paroxysm from time of prescription then change; if the least amelioration either in paroxysm or apyrexia, wait. Also, if you find *simillimum*, no return ever occurs.

Dr. Baylies—Disease is often in *strata*, and a remedy will be a *simillimum* to that *stratum* only.

Section 238 was then read.

Section 238. When a single dose of the appropriate remedy has destroyed several paroxysms, and manifestly restored health, and notwithstanding which, indications of a fresh attack are seen some time after, then only can and ought the same remedy to be repeated, provided the totality of the symptoms is still the same. But this return of the same fever, after an interval of health, is not possible, except when the cause which excited the malady in the first instance still exercises its influence upon the convalescent, as occurs in marshy countries. In such a case a permanent cure is soldom effected but by removing the patient from the exciting cause, and advising him to go and reside in a mountainous district, if that which attacked him was a marsh intermittent fever.

The President—Observe the qualifications—patient manifestly restored by the appropriate remedy, but the *same* symptoms recur some time later, and it is a case of marsh intermittent. I have never seen precisely that combination in a large experience. Either the remedy has not been well chosen (in the cases I have seen), or the right potency has not at first been selected, or the patient has been indiscreet in his habits before his strength was regained, and thus produced relapse, or the second attack was a little different from the first. In my own practice I have never been obliged to send the patient away, but have cured him on the ground.

Dr. Baylies—I think so, and prefer my patient to remain, as I can then be certain of a cure.

Meeting adjourned. EMMA D. WILCOX, *Secretary*.

